# Oral Microbiome Analysis and Caries Risk Classification Using the Caries Management by Risk Assessment System in Pre-Orthodontic Patients

**DOI:** 10.3390/jcm14186464

**Published:** 2025-09-13

**Authors:** Isamu Kado, Ryo Kunimatsu, Yuma Koizumi, Yuki Yoshimi, Tomohiro Ogasawara, Fumika Abe, Shintaro Ohgashira, Shangwu Tsai, Kanako Okazaki, Kotaro Tanimoto

**Affiliations:** 1 Department of Orthodontics and Craniofacial Developmental Biology, Graduate School of Biomedical and Health Sciences, Hiroshima University, Hiroshima 734-8553, Japan; isamu-kado@hiroshima-u.ac.jp (I.K.); tkotaro@hiroshima-u.ac.jp (K.T.); 2 Department of Orthodontics, Division of Oral Health and Development, Hiroshima University Hospital, Hiroshima 734-8553, Japan; ykoizumi@hiroshima-u.ac.jp (Y.K.);

**Keywords:** orthodontics, CAMBRA, bacteria, oral microbiome

## Abstract

**Background/Objectives**: This study aimed to classify pre-orthodontic patients using the Caries Management by Risk Assessment (CAMBRA) system and clarify their oral characteristics and microbiome. **Methods:** At the Department of Orthodontics, Hiroshima University Hospital, 68 patients were included in this study. Clinical parameters, such as plaque control record, DMF index, and number of white spot lesions (WSLs), were obtained. Medical interviews and oral examinations were conducted according to the CAMBRA system, and participants were classified into four risk groups (Low, Moderate, High, and Extreme). The supragingival plaques and stimulated saliva were collected. A saliva test was performed to measure the saliva secretion volume, pH, buffering capacity, and bacterial culture. Microbial DNA was extracted from the stimulated saliva and plaque samples, and 16S rRNA metagenomic analysis was performed. For statistical analysis, the Kruskal–Wallis test was used. **Results:** Participants were classified into four CAMBRA risk groups, with many classified as the High group. The number of DMF teeth and WSLs were the highest in the Extreme group, which tended to have the worst oral hygiene habits. The saliva test results revealed that the Extreme group had the worst saliva secretion volume, buffering capacity, and *Streptococcus mutans* score, with statistically significant differences. Bacterial 16S metagenomic sequencing revealed that the genus *Fusobacterium* had the highest relative abundance in the saliva samples of the Low group, whereas the genus *Actinomyces* had the highest relative abundance in the Extreme group. **Conclusions:** In this single-center, cross-sectional study, CAMBRA risk classification accurately reflected the oral condition of pre-orthodontic patients.

## 1. Introduction

Orthodontic treatment dramatically improves the occlusion and quality of life of patients. However, orthodontic treatment can cause various environmental changes in the mouth, resulting in side effects, such as root resorption, temporomandibular disorders, and bacterial infectious diseases. During orthodontic treatment, the pH of the unstimulated saliva reportedly decreases [1]. The incidence of white spot lesions (WSLs) and dental caries has been reported to increase following attachment of fixed orthodontic appliances [2,3,4]. Moreover, orthodontic treatment has also been reported to adversely affect periodontopathogenic bacteria [5]. However, a previous study reported that the increase in the risk of caries or gingivitis during orthodontic treatment can be prevented using an appropriate preventive protocol [6]. Thus, orthodontists should prevent iatrogenic disorders caused by oral bacterial infections, especially dental caries, during orthodontic treatment. To achieve this, it is necessary to determine the susceptibility of each patient to dental caries during the initial examination. Accordingly, stratifying caries risk before appliance placement and identifying modifiable factors, such as saliva secretion and buffering capacity, cariogenic bacteria, and oral hygiene, are essential to guide preventive measures prior to treatment.

Although restorative and prosthodontic treatments can help recover tooth form and function, they cannot alter caries risk factors such as saliva properties, oral microbiome, and lifestyle habits [7]. A personalized risk assessment for the future occurrence of dental caries is important for its management. To date, many studies have been conducted to prevent the occurrence of dental caries and identify a suitable method for determining the risk of dental caries [8,9,10,11]. Two major caries risk assessment (CRA) systems have been extensively studied over a long period: the Cariogram from Sweden and Caries Management by Risk Assessment (CAMBRA) from the USA [12]. The CAMBRA system is a two-step process involving both the CRA and caries management method developed at the University of California, San Francisco [13]. This system is highly predictive of the future occurrence of dental caries at all ages, as supported by several previous studies [11,14,15,16]. The CAMBRA system can classify patients into four risk groups based on basic oral examinations, radiographic images, and medical questionnaires using the CRA form and can provide chemical therapy according to each risk level [14,15,17].

While many studies have described changes after appliance placement, evidence that systematically classified pre-orthodontic patients using CAMBRA integrating clinical parameters for guiding preventive procedures before appliance placement remains limited. Our study aimed to classify pre-orthodontic patients using CAMBRA, and clarify their oral characteristics and microbiome for preliminary procedures.

## 2. Materials and Methods

### 2.1. Ethics Statement

This study was approved by the Independent Ethics Committee of Hiroshima University Hospital, Hiroshima, Japan (No. E-2761). Informed consent was obtained from the participants’ parents or legal guardians as this study included individuals aged 18 years. All the methods were performed in accordance with the relevant guidelines and regulations.

### 2.2. Participants and Sample Size Estimation

Among the patients who visited the Department of Orthodontics, Hiroshima University Hospital from July 2022 to January 2023, 68 patients (46 females, 22 males: median age was 14.2 years) who consented to participate in this study were included in this study (Table 1). All the participants were of Asian ethnicity. We calculated the test power using G*Power 3.1 software (Heinrich Heine University, Düsseldorf, Germany) to check the degree of statistical power.

### 2.3. Grouping by CAMBRA and Sampling

All participants underwent routine oral examinations, including oral photography, facial photography, and radiography (cephalometric and panoramic), and a study model was obtained at the beginning of orthodontic treatment at the initial visit. Clinical parameters, such as Plaque control record (PCR), decayed, missing, filled (DMF) index, and number of white spot lesions (WSLs), were obtained and oral hygiene instructions were provided [18,19,20]. Dental plaque was disclosed using a plaque disclosing solution, and O’Leary PCR method was used: for each erupted tooth, four surfaces (mesial, distal, buccal, and lingual) were scored as plaque present/absent and PCR (%) was calculated [19]. Next, medical interviews and oral examinations were conducted according to the CAMBRA system, and the participants were classified into four risk groups: Low-, Moderate-, High-, and Extreme groups. The patient was most likely classified in the Low group when disease indicators were absent, very few or no risk factors were present, and protective factors prevailed. If the patient was not obviously at High or Extreme, and being at Low was doubtful, the patient should be allocated to the Moderate group. One or more disease indicators are most likely to indicate at least high risk. If hyposalivation also occurs, the patient is likely to be at extreme risk [21].

The participants were interviewed and scored according to their oral hygiene habits. Those who brushed their teeth properly after every meal were given a score of 1; those who always brushed their teeth before going to bed but not always on other occasions were given a score of 2; and those who sometimes went to bed without brushing their teeth were given a score of 3.

Supragingival plaque and stimulated saliva samples were collected. Before sampling, the participants were instructed to refrain from eating, smoking, and tooth brushing for at least 2 h and avoid using a mouthwash. Approximately 4 mL of stimulated saliva samples were collected using tasteless gum containing Checkbuf (Horiba, Kyoto, Japan) and divided for saliva testing and microbial analysis [22]. For plaque analysis, the sampling area was isolated with cotton rolls, and saliva was removed by general air drying. Supragingival plaque was collected using a sterilized dental explorer [5]. Saliva samples for the saliva test were immediately subjected to a saliva test and the other samples were pooled into a microcentrifuge tube containing 0.5 mL of phosphate-buffer saline (PBS) and stored immediately at −80 °C until the microbial analysis [23].

### 2.4. Saliva Test

The saliva secretion rate (mL/min) was calculated based on the total amount of stimulated saliva collected using a tasteless gum. Saliva pH was measured using a Checkbuf pH meter (Horiba, Kyoto, Japan), and the buffering capacity of the saliva was measured using the CAT21Buf risk test (Morita Co., Osaka, Japan) [24]. The bacterial counts of *Streptococcus mutans* (*S. mutans*) and *Lactobacillus* were measured using Dentocult-SM and Dentocult-LB (Aidian, Helsinki, Finland), respectively, cultured at 37 °C for 48 h, and evaluated on a 4-point scale for each test, calculating the bacterial score as CFUs (score 0: <10^5^ CFU, score 1: <10^6^ CFU, score 2: 10^6^–10^7^ CFU, score 3: >10^7^ CFU) [22,25]. *Candida albicans* was cultured in CHROMager Candida medium (CHROMager, Paris, France) for 48 h at 37 °C and colony growth was recorded on a three-tier semi-quantitative scale (score 0: none, score 1: light, score 2: heavy) according to the manufacturer’s instruction by the clinical laboratory technicians.

### 2.5. Bacterial 16S Metagenomic Sequencing

Microbial DNA was extracted from the stimulated saliva and plaque samples using QuickGene-Mini 480 and Tissue DNA Kit S (KURABO, Kurashiki, Japan). The V3–V4 hypervariable regions of 16S rRNA gene were amplified by amplicon polymerase chain reaction with the forward primer “TCGTCGGCAGCGTCAGATGTGTATAAGAGACAGCCTACGGGNGGCWGCAG” and the reverse primer “GTCTCGTGGGCTCGGAGATGTGTATAAGAGACAGGACTACHVGGGTATCTAATCC” (Hokkaido System Science Co., Hokkaido, Japan) [26]. After amplicon polymerase chain reaction, the Library was prepared using the Nextera XT Index Kit (Illumina. San Diego, CA, USA) [5,26]. The library was applied to the MiSeq sequencing platform. DNA Sequence data were analyzed using the microbial multiomics data science system QIIME2. Reads were denoised with DADA2 after trimming primer regions and subsequently merged to generate representative sequences. Alpha diversity was quantified using Shannon entropy and compared across the CAMBRA risk groups. Beta diversity was assessed using Weighted UniFrac distance matrices. Principal coordinates analysis (PCoA) was used for ordination. Group differences across CAMBRA risk groups were tested by permutational multivariate analysis of variance (PERMANOVA). Nucleotide sequence data reported are available in the DNA Data Bank of Japan (DDBJ: https://ddbj.nig.ac.jp/search/entry/bioproject/PRJDB20162 (accessed on 30 April 2025) Sequenced Read Archive under the project accession number PRJDB20162.

### 2.6. Statistical Analysis

Statistical analyses were conducted by QIIME2 and GraphPad PRISM version 10.6.0 (GraphPad Software Inc. San Diego, CA, USA). Continuous or ordinal outcomes are summarized as median. Differences among the risk groups were first assessed using the Kruskal–Wallis test (two-sided α = 0.05). When the omnibus Kruskal–Wallis test was significant, post hoc pairwise comparisons were performed using Dunn’s test with multiplicity-adjusted *p*-values. For microbiome beta diversity, the PERMANOVA test was performed using QIIME2.

## 3. Results

### 3.1. Power Estimation

The sample size was calculated using G*power 3.1 software (Heinrich Heine University, Düsseldorf, Germany). One-way analysis of variance was performed for the four groups, with a total sample size of 68, effect size of 0.5, a significance level of 5%, and a calculated power of 0.934.

### 3.2. Subject Grouping by CAMBRA

**Figure 1:**
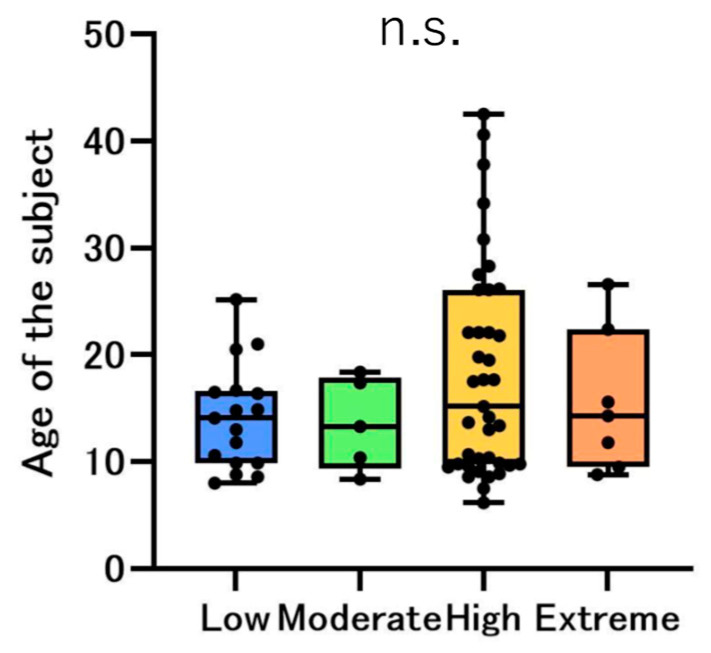
Age distribution of the participants by the risk group. The bar chart shows the median age of each risk group; blue, green, yellow, and orange represent the Low, Moderate, High, and Extreme risk groups, respectively. The black dots represent the distribution of each participant. No significant differences were observed between the risk groups (n.s.: not significant).

**Figure 2:**
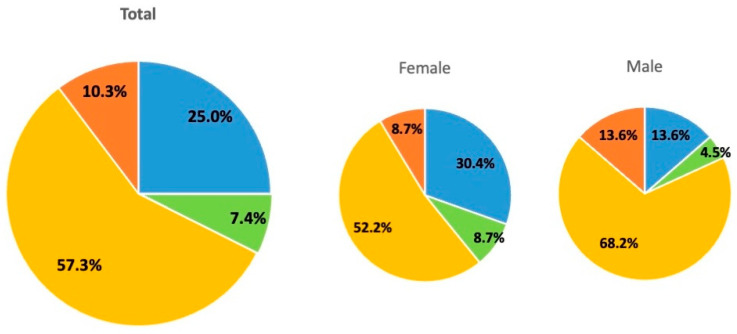
Percentage of risk groups by sex. Pie charts show the proportion of female and male participants classified into the four risk groups. The Low group is represented in blue, the Moderate group in green, the High group in yellow, and the Extreme group in red, each distinguished by color.

The participants were classified using CAMBRA (Table 1). There were 17 (25.0%) patients in the Low group, five (7.4%) in the Moderate group, 39 (57.3%) in the High group, and seven (10.3%) in the Extreme group. When comparing the median age, no statistically significant differences were observed between the risk groups (Figure 1). Females comprised 30.4%, 8.7%, 52.2%, and 8.7% of the Low-, Moderate-, High-, and Extreme-risk groups, respectively. Males comprised 13.6%, 4.5%, 68.2%, and 13.6% of the Low-, Moderate-, High-, and Extreme-risk groups, respectively (Figure 2).

### 3.3. Oral Examinations

**Figure 3:**
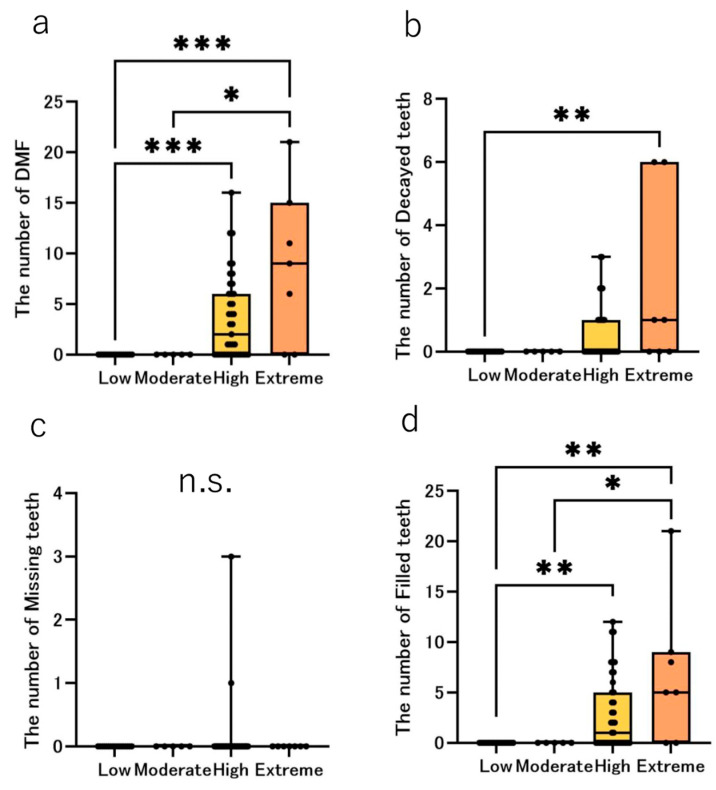
The median DMF index by risk groups. The bar chart shows the median number of decayed, missing, and filled (DMF) indices, where blue, green, yellow, and orange represent the Low, Moderate, High, and Extreme groups, respectively. The black dots represent the distribution of each participant. Comparison of the median number of (**a**) DMF, (**b**) D, (**c**) M, and (**d**) F teeth using the Kruskal–Wallis test. Significant differences are indicated by * *p* < 0.05, ** *p* < 0.01, *** *p* < 0.001 and n.s.: not significant.

The median number of DMF teeth was 0 in the Low- and Moderate groups, but was 2.0 in the High group and 9.0 in the extreme group (Figure 3a). Differences among groups were significant (*p* < 0.001). Dunn’s post hoc tests showed Extreme > Low (*p*_adj_ = 0.0009), High > Low (*p*_adj_ = 0.0007) and Extreme > Moderate (*p*_adj_ = 0.0225). The median number of decayed teeth was 0 in the Low, Moderate and High groups, but was 1.0 in the Extreme groups and the differences between groups were significant (*p* = 0.0064) (Figure 3b). Dunn’s post hoc tests showed Extreme > Low (*p*_adj_ = 0.0097). The median number of missing teeth was 0 in all the groups. No significant differences were observed among the risk groups (Figure 3c). The median number of filled teeth was 0 in the Low and Moderate groups, but was 1.0 and 5.0 in the High and Extreme groups, respectively. Differences among groups were significant (*p* = 0.002). Dunn’s post hoc tests showed Extreme > Low (*p*_adj_ = 0.0002), High > Low (*p*_adj_ = 0.0041) and Extreme > Moderate (*p*_adj_ = 0.0359). (Figure 3d).

**Figure 4:**
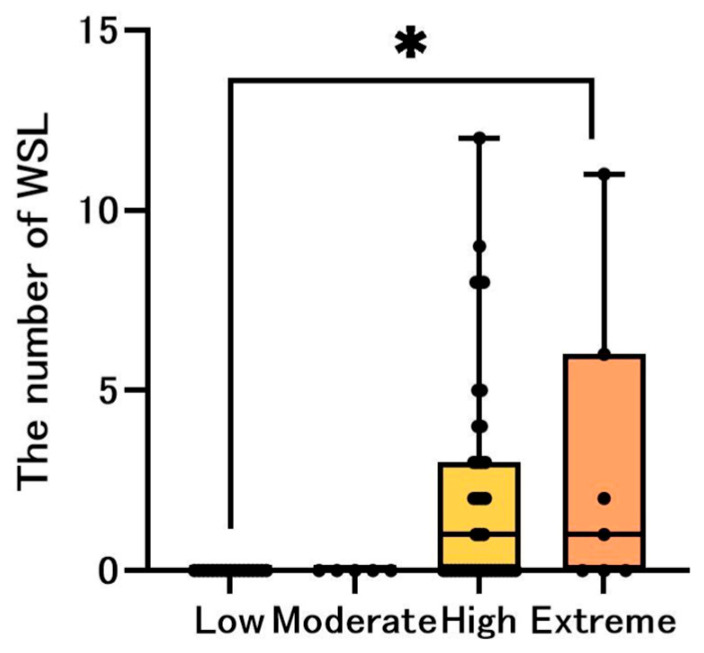
The median number of WSLs by the risk groups. The bar chart shows the median number of white spot lesions (WSLs), where blue, green, yellow, and orange represent the Low, Moderate, High, and Extreme groups, respectively. The black dots represent the distribution of each participant. Comparison of the median number of WSL using the Kruskal–Wallis test. Significant differences are indicated by * *p* < 0.05.

In this study, we considered a visible white spot that did not require restoration to be a WSL [18]. The median number of WSLs was 0 in the Low and Moderate groups but was 1.0 in the High and Extreme groups. Difference among groups was significant (*p* = 0.009). Dunn’s post hoc tests showed Extreme > Low (*p*_adj_ = 0.0023) (Figure 4).

**Figure 5:**
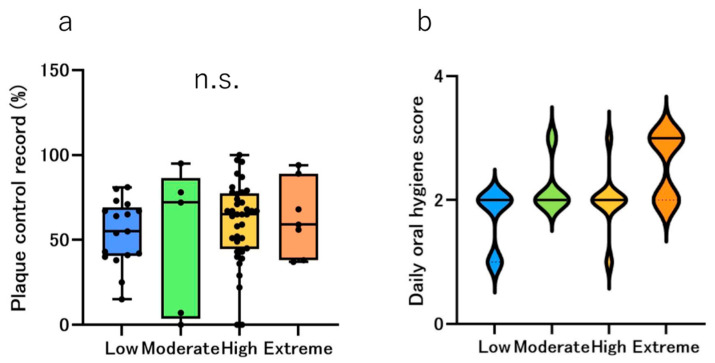
Oral hygiene condition by risk groups. The bar chart shows the median number of plaque control record (**a**) and the violin chart shows the oral hygiene habits (**b**), where blue represents the low group, green represents the moderate group, yellow represents the high group, and orange represents the extreme group. The black dots in the bar chart represent the distribution of each participant. However, the differences were not statistically significant (n.s.: not significant).

The plaque control record (PCR) was performed using a dental plaque-disclosing agent. The median PCR scores were 55%, 72%, 65%, and 59% in the Low, Moderate, High, and Extreme groups, respectively. However, no statistically significant difference was observed among all the risk groups upon multiple comparisons (Figure 5a).

The results revealed that the Low and Extreme groups had the best and worst oral hygiene habits, respectively (Figure 5b).

### 3.4. Saliva and Bacterial Culture Test

**Figure 6:**
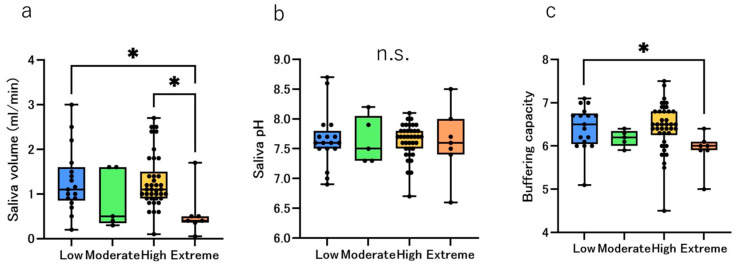
Salivary properties by risk groups. The bar chart shows the salivary properties; blue, green, yellow, and orange represents the Low, Moderate, High, and Extreme groups, respectively. The black dots represent the distribution of each participant. Comparison of the median number of (**a**) stimulated salivary secretion volumes, (**b**) saliva pH, and (**c**) salivary buffering capacity using the Kruskal–Wallis test. Significant differences are indicated by * *p* < 0.05 and n.s.: not significant.

The median salivary secretion volumes (mL/min) were 1.1, 0.5, 1.1 and 0.4 in the Low, Moderate, High and Extreme groups, respectively. Difference among groups was significant (*p* = 0.0157). Dunn’s post hoc tests showed Extreme > Low (*p*_adj_ = 0.0497) and Extreme > High (*p*_adj_ = 0.0159). (Figure 6a). The stimulated salivary pH was measured, and the results are shown in Figure 6b. The pH values were 7.6, 7.4, 7.7 and 7.5 in the Low, Moderate, High and Extreme groups, respectively, but the differences were small and not statistically significant. The buffering capacity of the stimulated saliva is shown in Figure 6c. Although the results differed from those for stimulated salivary pH, with stimulated salivary values decreasing with increased caries risk. The lower salivary buffering capacity of the Extreme group was statistically significant compared to the Low group. The medians of buffering capacity were 6.5, 6.2, 6.5, and 5.9 in the Low, Moderate, High, and Extreme groups, respectively. Difference among groups was significant (*p* = 0.0193). Dunn’s post hoc tests showed Extreme > Low (*p*_adj_ = 0.0301). No statistically significant difference was observed in the Moderate group, whose median value was lower than that of the Low group, owing to the small sample size.

**Figure 7:**
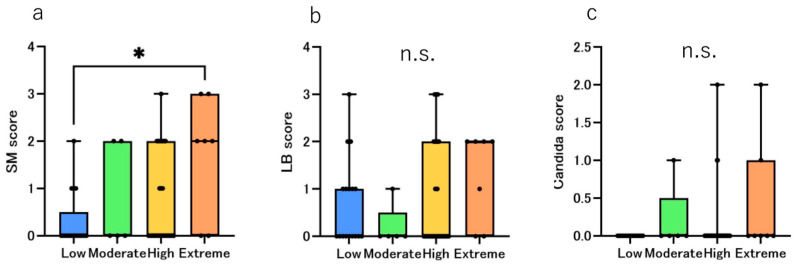
The results of the bacterial culture score by risk groups. The bar chart shows the median score of the bacterial culture test, where blue, green, yellow, and orange represent the Low, Moderate, High, and extreme groups, respectively. The black dots represent the distribution of each participant. Comparison of the median scores of (**a**) *Streptococcus mutans*, (**b**) *Lactobacillus*, and (**c**) *Candida albicans* using the Kruskal–Wallis test. Significant differences are indicated by * *p* < 0.05 and n.s.: not significant.

The *Streptococcus mutans* scores are shown in Figure 7a. The median *Streptococcus mutans* score in the Low, Moderate, and High groups was 0 but it was 2.0 in the Extreme groups. Difference among groups was significant (*p* = 0.0416). Dunn’s post hoc tests showed Extreme > Low (*p*_adj_ = 0.0258). This indicates a difference of more than 10 times in colony forming units (CFUs). The *Lactobacillus* scores are shown in Figure 7b. The median LB score in the Low, Moderate, and High was 0 but it was 2.0 in the Extreme groups. Although there were certain differences between the Extreme group and the other groups, the differences were not statistically significant. The results of the *Candida albicans* score are shown in Figure 7c. The median candida score was 0 in all the groups and no statistically significant differences were observed.

### 3.5. Bacterial 16S Metagenomic Sequencing

**Figure 8:**
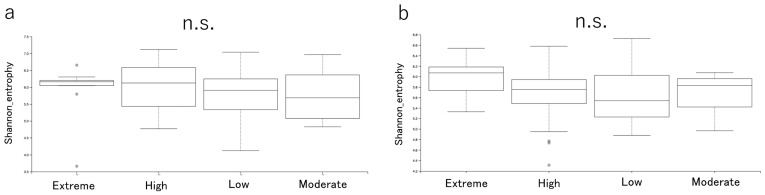
Alpha diversity (Shannon) across CAMBRA risk groups. Boxplots show median, interquartile range and IQR whiskers. (**a**) Plaque samples: Kruskal–Wallis H = 2.631, *p* = 0.452. (**b**) Saliva samples: Kruskal–Wallis H = 3.001, *p* = 0.391, n.s. indicates that the result was not significant.

**Figure 9:**
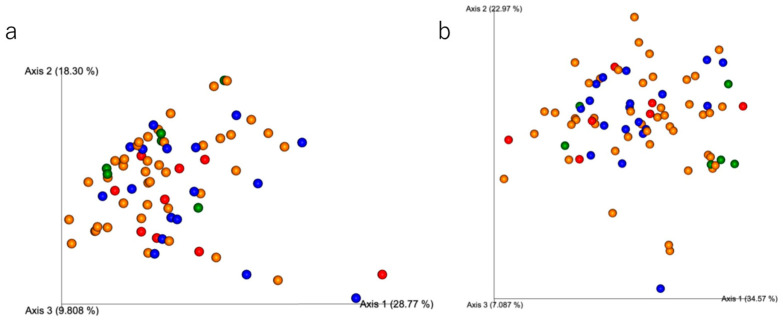
Beta diversity (weighted UniFrac PCoA) across CAMBRA risk groups. (**a**) Plaque samples. Global PERMANOVA: pseudo-F = 1.1618, *p* = 0.083. No global separation among groups was detected. (**b**) Saliva samples. Global PERMANOVA: pseudo-F = 0.9385, *p* = 0.502. No global separation among groups was detected. Each dot represents a sample and the Low group is represented in blue, the Moderate group in green, the High group in yellow, and the Extreme group in red, each distinguished by color.

**Figure 10:**
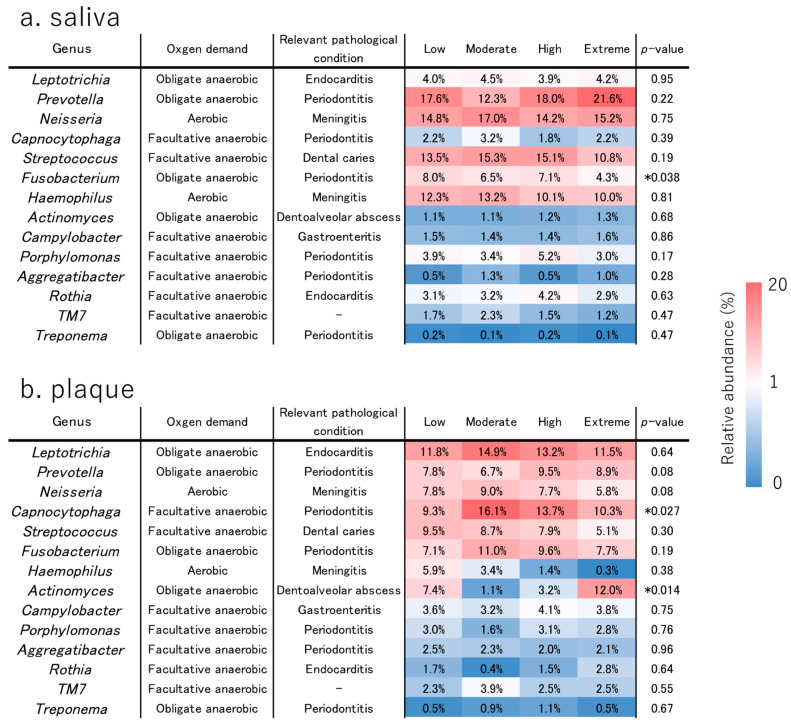
Bacterial population structure at the genus level. Heat maps of the relative abundance of bacteria in the supragingival saliva (**a**) and plaque (**b**) samples of each risk group. The oxygen demands and relevant pathological conditions of the pathogenic bacteria are described. The Kruskal–Wallis test was used, and significant differences are indicated by * *p* < 0.05.

Shannon alpha diversity did not differ among the CAMBRA risk groups in the plaque samples (Kruskal–Wallis H = 2.631, *p* = 0.452) (Figure 8a). Similarly, Shannon alpha diversity did not differ among groups in the saliva samples (Kruskal–Wallis H = 3.001, *p* = 0.391) (Figure 8b). Beta diversity (PCoA of weighted UniFrac distance) of plaque samples did not show distinct clustering by risk groups (pseudo-F = 1.1618, *p* = 0.083) (Figure 9a). Similarly, beta diversity (PCoA of weighted UniFrac distance) of the saliva samples showed no variation by the risk groups (pseudo-F = 0.9385, *p* = 0.502) (Figure 9b). The bacterial structure at the genus level is shown in Figure 10. The relative abundance of oral bacteria was compared among the risk groups. In the stimulated saliva data, the genus *Fusobacterium*, which includes species of periodontopathogenic bacteria, had the highest relative abundance in the Low group, and this result was statistically significant (Figure 10a). The relative abundance ratios of in the Extreme group were the highest for *Prevotella* and lowest for *Streptococcus*, but the differences were not statistically significant. Considering the supragingival plaque data, the genus *Actinomyces*, which includes species of cariogenic bacteria, had the highest relative abundance in the Extreme-risk group; this result was statistically significant (Figure 10b). The microbiome differed between saliva and plaque samples, with the genera *Leptotrichia*, *Capnocytophaga*, and *Actinomyces* being more abundant in the plaque samples, and *Prevotella*, *Neiserria*, and *Haemophilus* being more abundant in the saliva samples.

## 4. Discussion

### 4.1. Distribution by the Risk Groups and Oral Hygiene Status

Doméjean et al. performed a retrospective study validating the CDA CAMBRA system in 2011 [16]. Within the 6-year inclusion period of that study, 12,954 patients underwent a baseline CRA, and the overall caries risk was determined: 15.5%, 21.9%, 60.5%, and 2.1% were classified as low, moderate, high, and extreme risk, respectively [16]. Compared to the findings of that retrospective study, the proportion of the Low group was higher; conversely, the proportion of the Extreme group was also higher in this study (Figure 2). In the CAMBRA system, the presence of any disease indicator and hyposalivation automatically establishes an extreme risk; therefore, it is possible that the proportion of participants in the Extreme group was high because many individuals in this population were determined to have hyposalivation for reasons, such as nervousness in the special environment of visiting an orthodontist for orthodontic treatment. In this study, 100% of the participants were Asian (Japanese), but in the previous study, Asians comprised 16.1% of the participants. Therefore, racial composition may have also influenced the differences in the results.

However, when comparing the median age by risk category, no statistically significant differences were found, suggesting no influence of medication on hyposalivation with increased age (Figure 1). The results of the distribution by risk group between females and males demonstrated that the combined proportion of those in the High and Extreme groups was 60.9% and 81.8% for females and males, respectively. Our previous research showed that males have poorer oral hygiene than females, and the sex difference observed in this study is consistent with these results [22]. Regarding the oral hygiene status and habits, PCR showed the highest value in the Extreme group; however, no statistically significant difference was observed between the groups (Figure 5a). The violin chart revealed that the Extreme group had the worst oral hygiene habits (Figure 5b). These results revealed that the Extreme group had poorer oral hygiene habits than the other groups. Orthodontic treatment is essentially considered in patients who are capable of maintaining good oral hygiene. Therefore, in our department, we provide oral hygiene instructions and begin orthodontic treatment only after improvement in the oral hygiene. It is possible that toothbrushing instructions provided at orthodontic clinics could improve oral hygiene [27,28]. Numerous studies have documented the importance of maintaining and improving daily oral hygiene in preventing the development of caries during orthodontic treatment [29].

### 4.2. Intraoral Conditions

Our study comparing the number of DMF teeth and WSLs by risk groups revealed that all items scored 0 points in the Low/Moderate groups, whereas all the items except the number of missing teeth were positive in the High/Extreme groups. In the CAMBRA system, the presence of any disease indicators (1; Visible cavities or radiographic penetration of the dentin, 2; Radiographic approximal enamel lesions (not in dentin), 3; White spots on smooth surfaces, 4; Restorations last 3 years) automatically indicates high/extreme [21]. With this CAMBRA algorithm, it is natural that the number of DMF teeth and WSLs would be higher in the High/Extreme groups. However, it should be noted that the number of missing teeth was zero in all the risk groups. Since the population in this study included patients starting orthodontic treatment, we speculate that the likelihood of including patients with extremely poor oral hygiene was lower than that in the general population. A previous report stated that the likelihood of the presence of untreated dental caries was lower in participants who received orthodontic treatment than in those who did not [30]. Choi et al. reported that the number of active dental caries was lower in patients who received orthodontic treatment (0.43) than in those who did not (0.74). Thus, we believe there were no major dental issues in patients who were judged to be at lower risk than those in the High group in this study; however, since the number of decayed teeth in the Extreme group (Figure 3b) was nearly three times that of the population not receiving orthodontic treatment [30], immediate initiation of orthodontic treatment should be postponed until improvement in the oral environment of the participants. Special attention is required not only for patients in the Extreme group, but also for patients in the Low group. Thorough check-ups at each visit are essential as indicated by the high incidence of new dental caries (23.6%) observed during follow-up in this study.

### 4.3. Salivary Properties and Bacterial Culture Test

Saliva plays a crucial role in the oral environment. Saliva affects the incidence of dental caries by buffering and neutralizing the acids produced by cariogenic bacteria [31]. In this study, the saliva secretion volume was the lowest in the Extreme group, which was statistically significant; however, this was not surprising considering the CAMBRA algorithm (Figure 6a). The pH of the saliva itself was the lowest in the Extreme group, but this was not statistically significant (Figure 6b). In contrast, the saliva buffering capacity was significantly lower in the Extreme group than in the Low group (Figure 6c). Our previous study revealed that the salivary buffering capacity is lower in women than in men and decreases with age [22]; however, the results of this study revealed no age differences between the groups, indicating that the differences are based on the risk classification itself. Another study reported that the salivary buffering capacity of caries-free children was high, whereas that of children with numerous active caries was low [32]. The results of culturing the collected saliva revealed that the highest number of colonies was detected in the Extreme group for all three species: *Streptococcus mutans*, *Lactobacillus* and *Candida albicans*; however, only *Streptococcus mutans* showed a statistically significant difference. The *streptococcus mutans* score was significantly higher in the Extreme group than that in the Low group (Figure 7a). Both *Streptococcus mutans* and *Lactobacillus* are involved in the development and progression of dental caries and are problematic for patients undergoing orthodontic treatment [33]. Especially *Streptococcus mutans*, which is a major etiological agent of human dental caries, primarily resides in biofilms that form on the tooth surfaces [34,35,36]. Thus, the Extreme group was at a much higher risk of developing dental caries than the other groups owing to not only hyposalivation but also low salivary buffering capacity and high *Streptococcus mutans* detection rate. Although our cohort included pre-orthodontic patients, studies during orthodontic treatment have demonstrated shifts in the salivary/plaque environment, changes in stimulated flow rate and buffering/pH, and increase in salivary *Streptococcus mutans* and *Lactobacillus*, which underscores the value of baseline risk stratification before appliance placement, particularly in the Extreme group [37].

### 4.4. Microbiome of Saliva and Plaque

The human mouth harbors more than 700 bacterial species, constituting one of the most diverse bacterial communities in the body [5,38]. The mouth comprises complex bacterial structures of hard and soft tissue, demonstrating unique variations in the oral microbiome [39,40].

With the widespread use of next-generation sequencing, research on oral microbiota has become more common; however, there have been no reports on differences in the oral microbiota according to the CAMBRA classification, especially in populations starting orthodontic treatment. In this study, based on the results of metagenomic analysis, we compared the relative abundance ratios of bacteria that may cause pathology in the human body according to the risk groups. The microbiome varied between the saliva and plaque samples, which is consistent with the results of previous reports [5,39,40,41]. Notably, the taxonomic patterns we observed fit established saliva-plaque niche differences and ecological selection. Saliva represents a shed, mixed community, whereas dental plaque is a surface-attached biofilm that consistently differs from saliva and often enriches *Actinomyces* [42,43,44]. In our cohort, the Extreme group showed lower salivary flow and buffering capacity and higher *Streptococcus mutans* (Figure 7a). These features indicate more frequent and prolonged pH drops, providing ecological pressure that favors acidogenic bacteria such as *Actinomyces* within plaque, consistent with its dominance in the Extreme plaque in our data [45,46]. Conversely, salivary *Fusobacterium*, a well-established bridging organism that mediates co-adhesion between early and late colonizers, can be relatively higher in caries free saliva, and thus does not necessarily indicate caries activity [44,47,48]. This aligns with higher abundance in our Low group saliva. In saliva, the genus *Fusobacterium* exhibited the highest relative abundance in the Low group, consistent with a prior report in which salivary *Fusobacterium* was higher in groups other than the severe caries group [49]. In plaque, the genus *Actinomyces* exhibited the highest relative abundance in the Extreme group (statistically significant), consistent with prior evidence linking *Actinomyces* to caries progression and dentoalveolar abscesses [45,50].

Although 16S rRNA profiling did not exhibit the highest relative abundance of the genus *Streptococcus* in the Extreme group in either saliva or plaque (Figure 10), the culture-based detection rate of *Streptococcus mutans* was highest in the Extreme group (Figure 7a), indicating a greater absolute burden of viable *S. mutans. Actinomyces* exhibited the highest relative abundance in the Extreme group plaque. Taken together, these complementary findings are coherent with a more acidogenic plaque milieu that may contribute to caries development in the Extreme group.

Alpha and beta diversity did not differ globally between the CAMBRA risk groups, yet niche-linked taxa did: *Actinomyces* was higher in the Extreme risk group plaque and *Fusobacterium* was higher in Low risk group saliva. This indicates taxon-specific shifts under ecological selection without a community-wide restructuring; genus level 16S analysis and the use of weighted UniFrac may further attenuate detection of subtle compositional changes, while culturing still captured a higher viable *S. mutans* burden in Extreme group.

### 4.5. Limitations

The limitation of this study is that the number of participants varied based on the risk classification, which made comparison between the groups challenging. In addition, this was a single-center, cross-sectional cohort of Japanese pre-orthodontic patients, which limits external validity to other age groups, ethnicities, as well as to populations not seeking orthodontic care and to patients undergoing active orthodontic treatment. Furthermore, metagenomic sequencing did not involve full-length 16S rRNA analysis, but instead focused on the V3-V4 region, meaning that the bacteria could not be identified at the species level.

## 5. Conclusions

In this study, CAMBRA risk classification accurately reflects the oral condition of pre-orthodontic patients. CAMBRA can be considered an effective screening method at the start of treatment. In particular, special consideration is warranted in all aspects such as oral hygiene habits, number of cariogenic bacteria, and saliva buffering capacity for the Extreme group.

## Figures and Tables

**Table 1:** Participants’ characteristics by risk groups. Number of patients, population, sex and age.

		Low			Moderate			High			Extreme	
	Female	Male	Total	Female	Male	Total	Female	Male	Total	Female	Male	Total
Number of patients	14	3	17	4	1	5	24	15	39	4	3	7
Population			25.0% (17/68)			7.4% (5/68)			57.3% (39/68)			10.3% (7/68)
Age (median)			14.1			13.3			15.2			14.3

## Data Availability

Nucleotide sequence data reported are available in the DNA Data Bank of Japan (DDBJ) Sequenced Read Archive under the project accession number PRJDB20162.

## Abbreviations

The following abbreviations are used in this manuscript:

CAMBRA	Caries Management by Risk Assessment
DMF	decayed, missing, filled
WSL	white spot lesion
CRA	caries risk assessment
PCR	plaque control record
CFU	colony forming unit
